# Editorial: The 4th international expert forum on the public health and environmental impacts of cellular and wireless radiation exposure 2024

**DOI:** 10.3389/fpubh.2026.1856852

**Published:** 2026-06-10

**Authors:** Paul Ben Ishai, Tom Butler, Devra Davis, Dariusz Leszczynski, Hugh Taylor

**Affiliations:** 1 Department of Physics, Ariel University, Ariel, Israel; 2 Cork University Business School, University College, Cork, Ireland; 3 Environmental Health Trust, Washington, DC, United States; 4 University of Helsinki, Helsinki, Finland; 5 Yale School of Medicine, New Haven, CT, United States

**Keywords:** biological effect, electromagnetic field (EMF), policy, public health, wireless and mobile technology

Wireless communication technologies are ubiquitous and ever-present, whether on the person, in homes, schools, transport systems, and across all occupational environments. Hence, humans and the environments in which they live and work are now exposed to a background of anthropogenic RF-EMF that differs qualitatively and quantitatively from naturally occurring sources (Lin). Successive generations of wireless technologies have increased network density, carrier frequencies, and the complexity of polarized and pulsed signal modulation (Panagopoulos et al.). However, policymakers continue to rely on scientific findings and outdated assumptions developed between the 1950s and the 1980s, most notably the premise that biological harm arises only through tissue heating or thermal effects (Lin; Frank; Héroux; Levitt et al.).

This Research Topic on the public health and environmental risks of cellular and wireless radiation is the culmination of the recent “*Expert Forum on the General Impact of Cellular and Wireless Radiation Exposure 2024*,” held at Yale Medical School, from 4 to 6 June 2024. It brings together 26 authors in 10 studies that take stock of the extant research findings on: (1) the association between RF-EMF exposures and adverse consequences for human health and the environment (Levitt et al.; Weller et al.); the inadequacy of health and safety protections and policies, and the need for and emergence of a paradigm shift from an unlikely source (Lin); the limitations of industry-oriented U.S. policymaking (Scarato); the flaws in epidemiological methods in industry research, standards, and guideline setting (doi: 10.3389/fpubh.2025.1559868); The socio-historical origins of the development of a “gulf” between institutional and independent scientists (Héroux); and the fundamental biological mechanisms triggered by anthropogenic EMFs that produce the adverse biological effects observed (Panagopoulos et al.). This Research Topic also presents three original research articles that shed new light on (a) prenatal RF-EMF exposure and its implications for early and subsequent development in rats (Altun and Kaplan), (b) neurodegeneration from high-energy light (an EMF) in insects, with clear implications for humans (Piacenti-Silva et al.), and (c) the alteration of human blood flow due to smartphone exposure, as observed in an N-of-1 study, with serious implications for cardiovascular and autonomic health issues in the broader population (Brown and Biebrich). Thus, this Research Topic is timely in that it aims to critically assess extant research findings on the association between exposure to RF-EMF and adverse biological and environmental effects, identify points of convergence and divergence therein that lead to uncertainty about the risks and the related institutionalization of inadequate standards and guidelines, which in turn is reflected in inadequate regulatory policies on human health, safety, and environmental protection.

The contributions to this Research Topic argue that the standards and guidelines on which regulators depend may lead to significant public health and environmental problems, as the speed of technological change and deployment has, by 2026, far outpaced the implementation of adequate, science- and evidence-based health and safety measures, with extant regulations being founded on the thermal-only paradigm (Lin; Frank; Scarato). The articles collected here challenge the legitimacy and suitability of the thermal-only paradigm, which was, with a few exceptions, reinforced by a recent series of systematic reviews (SRs) sponsored by the WHO EMF Project, which historically has been influenced by the ICNIRP and industry interests (1).

In Nicholas Steneck's seminal early history, *The Microwave Debate* (2), the author clearly identified several themes that are echoed in the articles by James Lin, Héroux, Frank, and Sacarato: (1) The dominance of the thermal-only paradigm and the rejection of non-thermal perspectives based more on ideological grounds than scientific evidence; (2) the role of industry in the shaping and influencing the institutionalization of health and safety standards, guidelines, and policies rather than valid public health concerns; (3) the persistent marginalization and outright rejection of findings on non-thermal effects; and (4) the exclusive focus on thermal-only effects by industry-aligned scientists. While the industry standards body, the IEEE, and its members held sway in the 1980s, they were joined by the ICNIRP in the 1990s, which also spawned and controlled the WHO EMF Project by the late 1990s. However, Lin points to an ongoing paradigm shift as military researchers in the United States investigate non-thermal effects.

**Figure 1:**
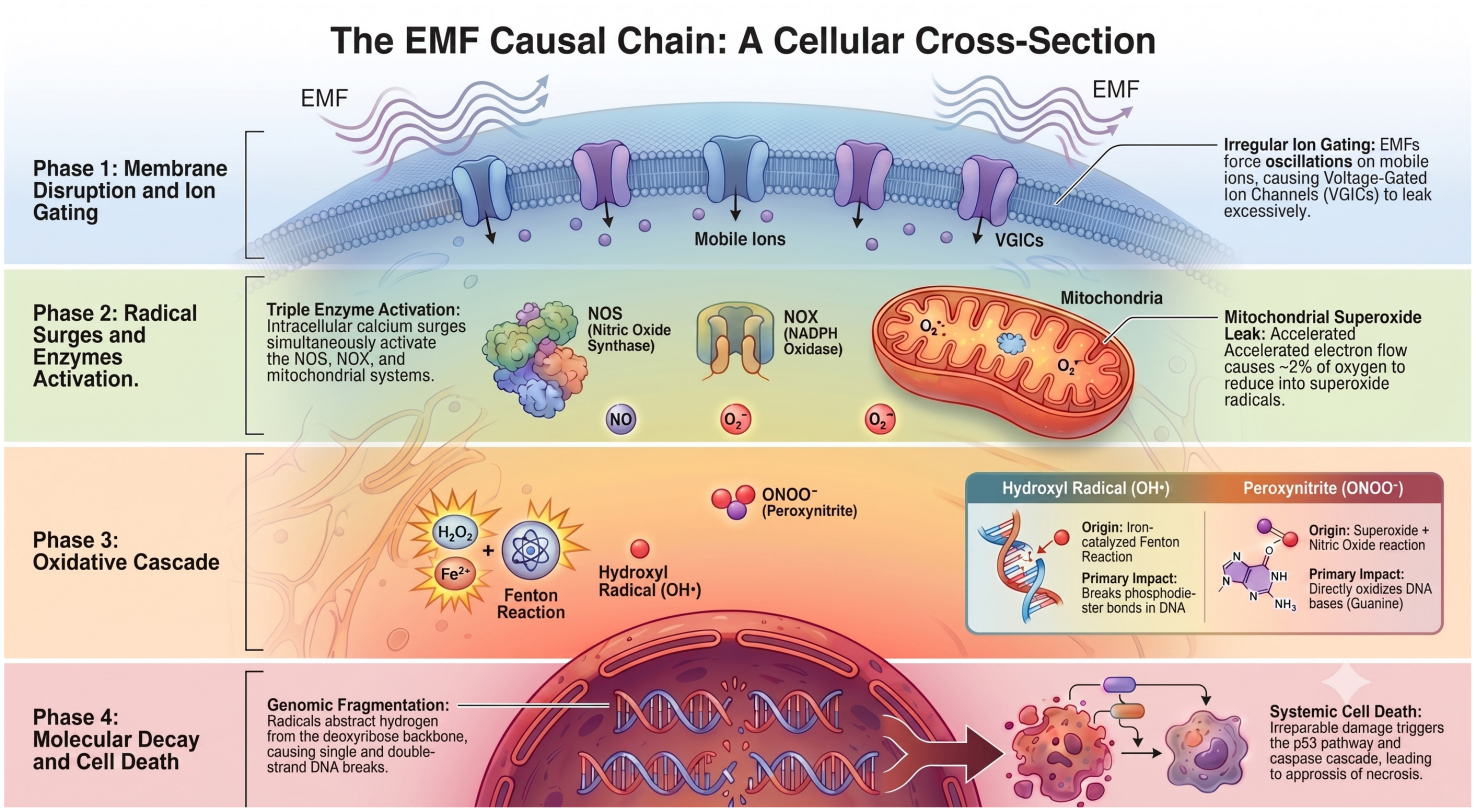
A schematic illustrating the causal chain of EMF interference with ion gates in cellular membranes.

In the preparation of the influential IEEE C95.l-1982 standard, Steneck (2) noted how “calcium efflux” was dismissed as a plausible mechanism by the U.S. engineering community of practice to help explain non-thermal biological effects. In this Research Topic, Lin and Levitt et al. revisit the issue in the context of oxidative stress, but it is Panagopoulos et al. who provide the “biological plausibility” required by Bradford Hill's viewpoints, as articulated by Frank. While others, such as Martin Pall, articulated the relationship between RF-EMF, voltage-gated calcium channels, calcium efflux, ROS, and oxidative stress, Panagopoulos et al. discuss the movement of calcium ions as a key element in their ion forced oscillation model. While they reference earlier work, Panagopoulos et al. emphasize that the disruption of calcium concentrations is a key trigger for subsequent oxidative stress (10). Formal identification of disrupted calcium concentrations following exposure to electromagnetic fields shifts the evidence from mere statistical association to a plausible causal explanation, directly challenging the “thermal-only” paradigm that has dominated public policy for decades. The argument that Panagopoulos et al. put forward is schematically illustrated in Figure 1. While Héroux exposes the specific technical flaws in physicist Robert Adair's influential dismissal of such mechanisms in the 1990s, Frank uses the Bradford Hill framework for inferring causation to argue that such dismissals are methodologically unsound. Furthermore, policymakers should not ignore biologically plausible pathways to harm reported by independent scientists, particularly as these have been of concern to scientists since the 1970s. Original research by Altun and Kaplan in this Research Topic demonstrates the sensitivity of the fetal brain to changes in calcium signaling during development. The authors note that electromagnetic fields can alter the expression of genes and proteins that depend on calcium homeostasis, contributing to neurodegenerative changes in the hypothalamus of exposed offspring (Altun and Kaplan). Collectively, these contributions frame calcium efflux and influx as vital evidence of the non-thermal, biologically active nature of RF-EMF, which current safety standards and guidelines continue to ignore (Lin; Panagopoulos et al.).

While the original research articles in this Research Topic provide additional evidence of adverse biological effects in insects (Piacenti-Silva et al.), animals (Altun and Kaplan), and humans (Brown and Biebrich), the comprehensive and scientifically rigorous research review by Weller et al. offers a timely correction to the less-than-rigorous findings of certain WHO EMF Project SRs, as noted by Lin in this Research Topic. Perhaps the only unbiased and scientifically rigorous of the WHO EMF SRs was that of Mevissen et al. (3), which reported findings of carcinogenicity and other disease endpoints. In terms of the association between RF-EMF exposure and cancers in humans, the negative findings of relevant WHO EMF SR by Karipidis et al. (4) were effectively rebutted by a contemporaneous, independent systematic review by Moon et al. (5), which found a significant association between RF-EMF exposure from mobile phone use and brain cancer, especially ipsilateral (same side) glioma, meningioma, and malignant brain tumors in long-term users with high cumulative use. It is notable, and heretofore unmentioned in the literature, that Karipidis et al. (4) cite the UK Biobank study's findings of no statistically significant association between RF-EMF and brain cancer, but those same authors fail to note the unequivocal findings of this large UK Biobank study:

“*Mobile phone use was significantly associated with higher risks of incident overall cancer [HR, 1.09; 95% confidence interval (CI): 1.06–1.12], nonmelanoma skin cancer (NMSC; HR, 1.08; 95% CI: 1.03–1.14), urinary tract cancer (HR, 1.18; 95% CI:1.05–1.32), and prostate cancer (HR, 1.19; 95% CI: 1.13–1.25) in men, and incident overall cancer (HR, 1.03; 95% CI: 1.00–1.06), NMSC (HR, 1.07; 95% CI: 1.01–1.13), and vulva cancer (HR, 1.74; 95% CI: 1.00–3.02) in women, but not with other cancers. Among mobile phone users, there was a dose–response relationship of length of mobile phone use with incident NMSC in men and women, and prostate cancer in men*” [(6), p. 88]. [SIC]

Had Karipidis et al. examined the UK Biobank findings in full, they would have discovered that the authors combined all types of brain cancer together and did not examine glioblastoma multiforme—the type most frequently associated with phone use in case-control studies (7, 8). It is significant that the most aggressive and fatal form of brain cancer, glioblastoma multiforme (GBM), more than doubled in incidence between 1994 and 2015, as indicated by Philips et al. (9), while other lower-grade tumors had decreased. This is, in retrospect, problematic for the WHO study, providing further evidence of systematic bias (Lin) and questionable value judgments (2).

Taken together, the contributions to this Research Topic suggest that historical disagreement reflects not only scientific uncertainty, human hubris, and individual and collective value judgments (2), along with institutional, methodological, and social influences that shape science and policy (Lin; Frank; Héroux; Scarato). While important knowledge gaps remain, particularly in long-term ecological research and exposure assessment, the current literature is sufficient to seriously question the continued reliance on the thermal-only paradigm to safeguard public health and the environment. The evidence presented in this Research Topic indicates plausible pathways for gradual, cumulative adverse effects on human health and wellbeing and on the ecosystem in which they exist and depend. For human populations, the principal risk lies in normalizing universal, continuous exposure to a biologically active, environmental toxin, while the policymaking and regulatory frameworks responsible for risk management lag behind scientific understanding of the health and environmental risks of RF-EMF. For ecosystems, the risk is compounded by stressors, such as habitat loss and climate change, which may amplify vulnerability to EMF exposure.

It is time that policy and regulation take into consideration the reduction of EMF exposure as a public and environmental health issue to be pursued. As clean air acts have forced changes in industry behavior, a similar regulatory approach is necessary to ensure scientifically reasonable levels of EMF exposure.

In this context, precaution should be understood not as opposition to technological development but rather as a governance principle aimed at aligning innovation with the protection of public and environmental health. This Research Topic does not close the debate on wireless RF-EMF and its biological effects. It does, however, clarify that the central question is no longer whether RF-EMF can interact with biological systems, but rather how the risks associated with such interactions and adverse consequences should be mitigated.
